# Supplementary material for: The role of perceived expertise and trustworthiness in research study and clinical trial recruitment: Perspectives of clinical research coordinators and African American and Black Caribbean patients
Source: PLoS One. 2023 Jun 21;18(6):e0275770. doi: 10.1371/journal.pone.0275770 (PMC10284411; doi:10.1371/journal.pone.0275770)
Supplement: S1 File — (DOCX) [file pone.0275770.s001.docx]

S1 File. Focus group questions for patients

1. Opening: Give us the name you’d like us to use, and tell us whether you’ve ever participated in a clinical trial or research study. (You don’t need to tell us what the study was focused on, especially if it would reveal anything you don’t want us to know about your health.)
2. Are there some types of research studies that you would be more likely to be willing to join?
3. Do you think your attitudes about participating in clinical trials or research studies are similar to other people in your family, community, or people who you know and like?
4. Please tell us about the experience you had when someone approached you about participating in a clinical trial or research study.
   1. Where were you when this happened?
   2. Did the person explain the study in a way you could understand? What kind of words did they use? If they used scientific terms, can you give some examples?
   3. How did the person make you feel? What did they do to make you feel that way? (tone of voice, eye contact, where they stood, how they sat)
5. If you could tell medical staff how they should interact with you or patients like you, what would you recommend? What would “good” communication look like? What kinds of “bad” communication practices should they avoid?
