# Supplementary material for: The role of perceived expertise and trustworthiness in research study and clinical trial recruitment: Perspectives of clinical research coordinators and African American and Black Caribbean patients
Source: PLoS One. 2023 Jun 21;18(6):e0275770. doi: 10.1371/journal.pone.0275770 (PMC10284411; doi:10.1371/journal.pone.0275770)
Supplement: S2 File — (DOCX) [file pone.0275770.s002.docx]

S2 File. Focus group questions for clinical research coordinators

1. Are there some types of research studies that Black Caribbean and/or African American communities more likely to be willing to join?
2. Are recruitment/retention challenges different in the African American community or the Black Caribbean community different from White/Hispanic communities? How are the challenges different between these two communities (AA vs BC)?
3. CRCs often indicate that it’s challenging to explain words like “research”, “clinical trials”, “experiments”, “placebos”, or “randomization.” Do you use different words/terminology to explain study participation when you’re talking to a member of Black Caribbean or African American communities?
4. Are there other things that you do to try to enhance the quality of your interactions with AA/BC patients as you explain a clinical trial? Are there certain ways that you use nonverbal communication that you find helpful? (For example, a certain tone of voice, how you orient your body to the patient, level of eye contact, etc.)
   1. Probe: Do you approach AA/BC women differently from men?
5. What do effective CRCs do when they talk about clinical trial participation with Black Caribbean patients? With African American patients? What does ineffective communication look like?
